# Clinical Approach to a Suspected Case of First Branchial Arch Syndrome

**DOI:** 10.1155/2014/506804

**Published:** 2014-01-12

**Authors:** Noboru Yamaguchi, Shiho Nakamura, Haruyoshi Yamaza, Soichiro Nishigaki, Keiji Masuda, Ken-ichi Yanagita, Kazuaki Nonaka

**Affiliations:** ^1^Section of Pediatric Dentistry, Division of Oral Health, Growth and Development, Faculty of Dental Science, Kyushu University, 3-1-1 Maidashi, Higashi-ku, Fukuoka 812-8582, Japan; ^2^Department of Pediatric Dentistry, Kyushu University Hospital, 3-1-1 Maidashi, Higashi-ku, Fukuoka 812-8582, Japan

## Abstract

First branchial arch syndrome is a congenital disorder characterized by a wide spectrum of anomalies in the first branchial arch, mainly affecting the lower jaw, ear, or mouth, during early embryonic development. We sought to confirm a suspected case of this syndrome by making differential diagnosis and taking an intensive clinical approach. A 12-year-6-month-old girl with a horizontally impacted left canine in the maxilla had the history of digital fusion in her hands and feet and has been suffering from hearing impairment of her left ear. To diagnose this case and make her careful treatment plan, we further carried out cephalometric analysis and mutation analysis. Her face looks like asymmetry and is not apparently symmetric by cephalometric analysis. Mutation analysis of the patient was conducted by direct DNA sequencing of the goosecoid gene, which is an excellent candidate for determination of hemifacial microsomia, but no changes in this gene were identified. We could not precisely diagnose this case as first branchial arch syndrome. However, certain observations in this case, including hearing impairment of the left ear, allow us to suspect this syndrome.

## 1. Introduction

The first and second branchial arch syndrome results in a wide spectrum of anomalies that encompass diverse, superimposed, and heterogeneous phenotypes within the so-called oculoauriculovertebral spectrum [1, 2]. As a part of this syndrome, the term hemifacial microsomia has been used to refer to patients with unilateral microtia, macrostomia, and failure of formation of the mandibular ramus and condyle [3]. The term first and second branchial arch syndrome imparts the erroneous impression that involvement is limited to facial structures, but in fact cardiac, renal, and skeletal anomalies may occur as well [4–6]. Poswillo [6], using an animal model of hemifacial microsomia, was able to show that destruction of differentiating tissues in the region of the ear and jaw by an expanding hematoma produced branchial arch dysplasia. The severity of this dysplasia was related to the degree of local destruction. It is important to note that extreme variability of expression of first and second branchial arch syndrome is characteristic. Autosomal dominant, autosomal recessive, and multifactorial models of inheritance are all possibilities to consider.

In this present study, we were not obviously able to diagnose this patient with first and second branchial arch syndrome. However, as we herein report, this case needed to be examined carefully and a treatment plan made, because of the findings made during the patient's first visit. This study was approved by the ethics committee of Kyushu University Faculty of Dental Science, and informed consent was granted by the parents.

## 2. Case Reports

A 12-year-6-month-old Japanese girl visited a private general dental office, where radiographic examination revealed horizontal impaction of her left maxillary canine. When she was referred to our university hospital and first visited us, her weight was 44.6 kg (average for her age group: 43.9 kg) and her height was 156.1 cm (average for her age group: 151.5 cm). She displayed mouth breathing as an oral habit. She had undergone repair of bilateral syndactyly between the 1st and 2nd digits, the 2nd and 3rd digits of her hands and feet, and had been suffering from hearing impairment of her left ear.

**Figure 1:**
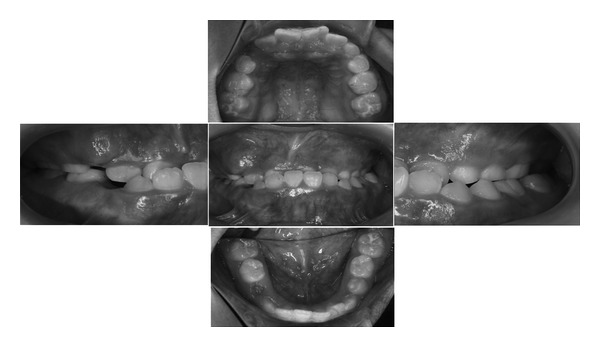
Intraoral photographs at the first visit of this patient.

**Figure 2:**
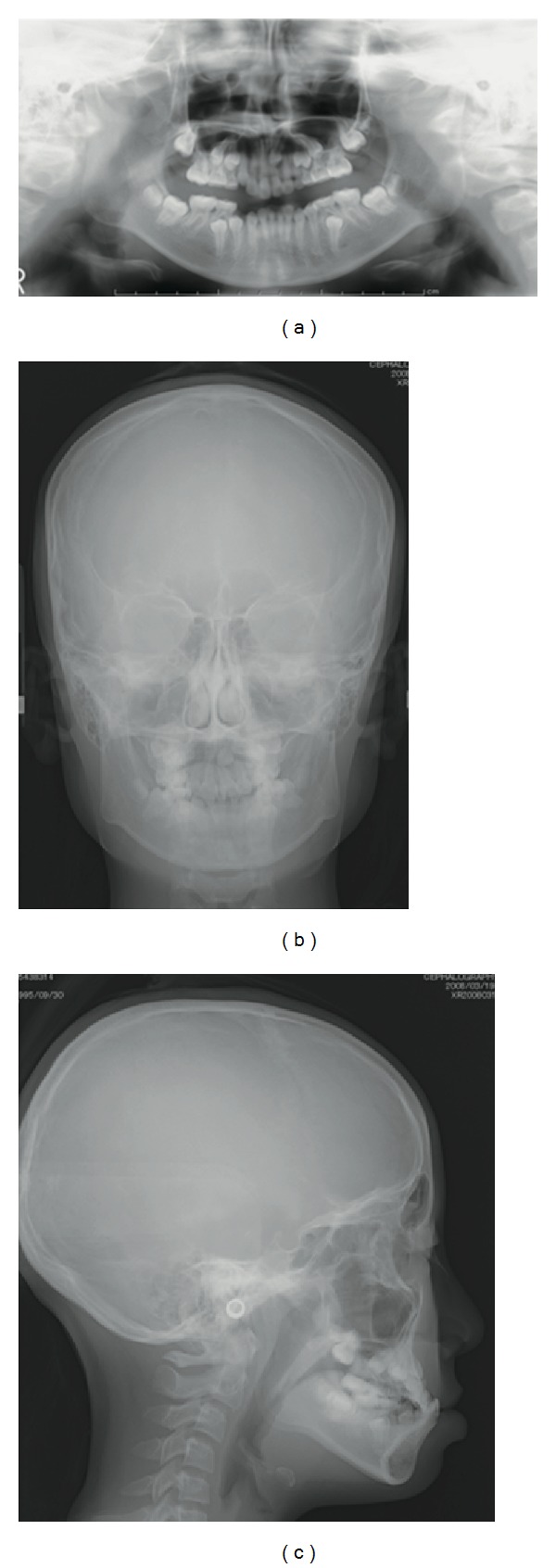
Radiographic appearance at the age of 12 years and 6 months: (a) panoramic radiograph, (b) frontal cephalometric radiograph, and (c) lateral cephalometric radiograph.

There were no relevant conditions or events in the medical history of her family members. Extraoral findings revealed the presence of mild facial asymmetry. The clinical examination showed that all erupted teeth were caries-free (Figure 1). An X-ray photographic examination showed that the left impacted canine existed between the left central incisor and the lateral incisor in the maxilla (Figure 2). Moreover, we obtained the further information from a CT scan indicating resorption of the root of the left central incisor.

### 2.1. Study Models Examination

**Figure 3:**
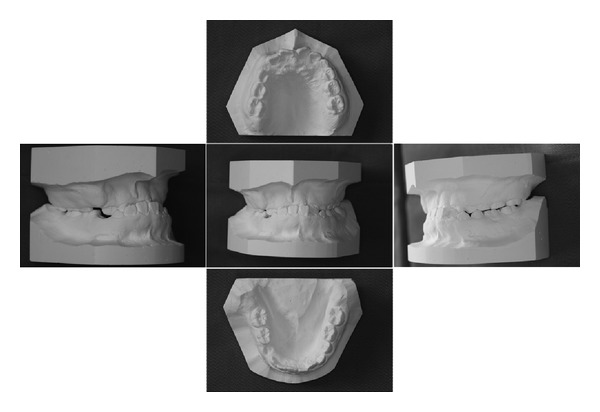
Study cast taken at the age of 12 years and 6 months.

The overbite was 6.76 mm and the overjet was −3.73 mm. The central position shifted to the left by 3.12 mm. The 1st molar occlusion type was class III at both sides. The results of space analysis were −3.10 mm in the upper side and +3.33 mm in the lower side (Figure 3). Other inspection items for the maxilla and mandible were within the range of the mean plus standard deviation.

### 2.2. Cephalometric Radiograph Analysis

For the cephalometric landmarks and standard values, data from the Japanese Society of Pediatric Dentistry were used [7]. All subjects in the database had normal occlusion and ranged in age from 8 years and 11 months to 13 years (mean age: 11 years old).

### 2.3. Mutation Analysis

**Figure 4:**
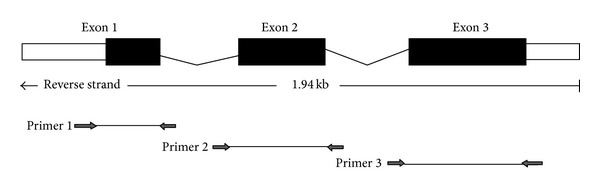
Analysis of human goosecoid (*GSC*) gene mutation in the 3 *GSC* exons. All 3 exons of the *GSC* gene were screened in this patient by sequence analysis using the fluorescent dideoxy terminator method and analyzed on an ABI 377 sequencer (Applied Biosystems). Intronic primers flanking each entire exon were designed from human genomic sequence (accession number AL121612) as described previously [9].

Following informed consent DNA was prepared from buccal epithelial cells by using a BuccalAmp DNA extraction kit (Epicentre, Madison, Wisconsin, USA) according to the manufacturer's protocol as described by Sasaki et al. [8]. All 3 exons of the goosecoid (*GSC*) gene were screened in this patient by sequence analysis using the fluorescent dideoxy terminator method and analyzed on an ABI 377 sequencer (Applied Biosystems, Figure 4). Intronic primers flanking each entire exon were designed based on the human genomic sequence (accession number AL121612) as described previously [9]. The primer sets were as follows: for exon 1, 5′-CCCACTTTAAGGCTCTGTCC-3′ (forward primer) and 5′-AATTAACCAACCGGCTCCAT-3′ (reverse primer); for exon 2, 5′-GCAGACGACTTCTAAGTGGAAGAG-3′ (forward) and 5′-TTCAACTTCCTGGGCCTAAA-3′ (reverse); and for exon 3, 5′-GCGCCTTTGATCTGAACTGT-3′ (forward) and 5′-TCGTCTGTCTGTGCAAGTCC-3′ (reverse).

### 2.4. Cephalometric Radiograph Analysis

The profilogram was compared with standard values to display the differences in skeletal features of the patient (Table 1). The angle of the A-B plane to the facial plane (−0.7°) and the interincisal angle (141.7°) were larger than the normal range of 2SD limits. The angle of the mandibular plane to the FH plane (25.6°) and the gonial angle (118.8°) were smaller than the normal range of 2SD limits.

**Figure 5:**
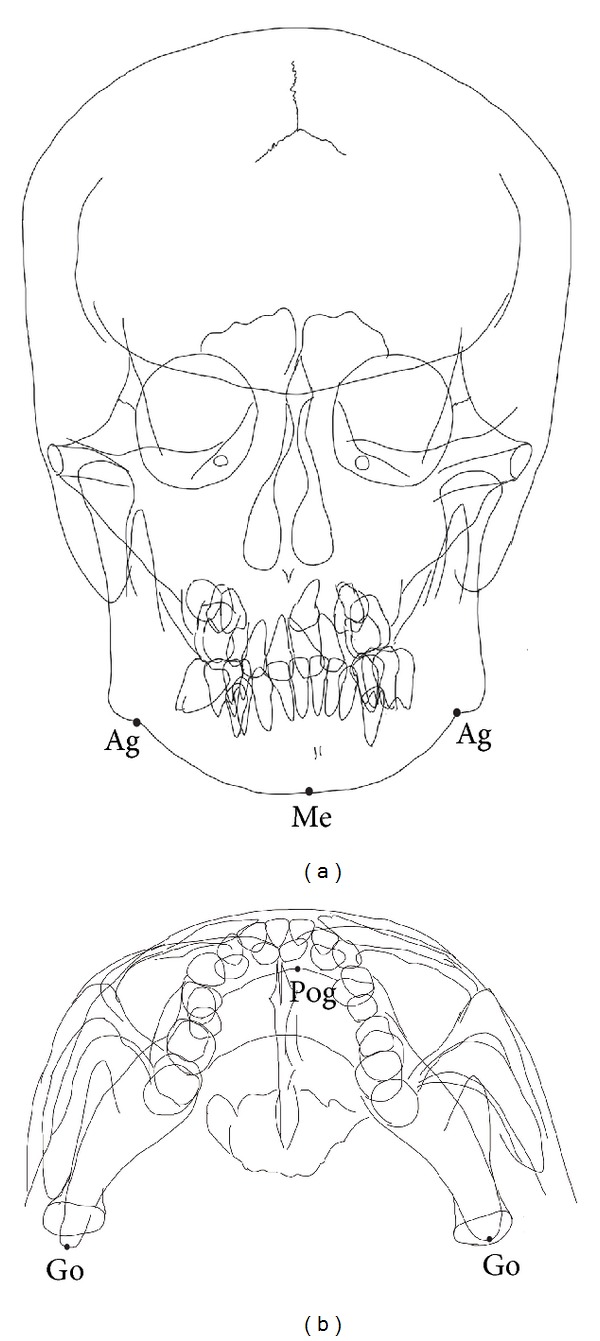
Trace of mandibular lines of this patient: (a) frontal cephalometric radiograph, (b) S-V cephalometric radiograph. Ag: antegonial tubercles; Mn: menton; Pog: pogonion; Go: gonion.

A trace using the frontal and the posterior-anterior cephalogram did not show obvious asymmetry (Figure 5). We used some cephalometric landmarks and measured the length between them. The length between the antegonial tubercles (Ag) and menton (Me) was 46.99 mm on the right side and 40.99 mm on the left side. Moreover, the length between pogonion (Pog) and gonion (Go) was 96.53 mm on the right side and 91.18 mm on the left.

### 2.5. Mutation Analysis

There was no mutation identified in any of the 3 exons of the *GSC* gene in this patient (data not shown). Using the same system, we analyzed the sequence of this gene in a healthy male adult as a control and confirmed no mutation in any of the 3 exons (data not shown).

## 3. Discussion

In the present case, extra-oral examination revealed no asymmetry of her mandibular bone. The cephalometric analysis did not show any obvious facial asymmetry, either (Figure 5). Hemifacial microsomia (HFM) is a common birth defect involving structures derived from the first and second branchial arches. Principal features include facial asymmetry, which is secondary to maxillary and mandibular hypoplasia, underdevelopment of the external ear preauricular skin tags or pits, and conductive hearing loss. The phenotype is extremely variable and may range from cases of isolated microtia to significant facial asymmetry. Tobiume et al. [10] reported an obvious case of first and second branchial arch syndrome in a patient whose profile and occlusion improved in response to orthognathic surgery.

As an approach towards identifying molecular pathways involved in ear and facial development, we examined the *GSC* gene in this case, because this locus is an excellent candidate for HFM based on mouse expression and phenotype data [11, 12]. The *GSC* gene comprises 3 exons. By exploiting the fact that human and mouse genomic organization, including exon-intron boundaries, is highly conserved [13], the human genomic sequence was deduced by comparison of human genomic clone AL121612 with the mouse cDNA sequence (accession number M85271). Mutation analysis was conducted by direct DNA sequencing of all 3 *GSC* exons in this patient, with no changes identified. However, it is not possible to rule out a complete inversion of the gene or the possibility of deletions or rearrangements outside the 9-kb genomic fragment analyzed that may give rise to the condition by a potential position effect [14].

A proportion of mice with a heterozygous disruption of their FGF8 gene showed a less severe phenotype than did those with a homozygous disruption, and the former phenotype was observed in the first branchial arch on only one side of the head [15]. Other models include the AP-2 knockout mouse, which exhibits severe craniofacial, neural tube, and skeletal defects [16], and homozygous loss of endothelin-1, which results in hypoplasia of the mandible and defects of the external and middle ear [17].

Regarding the bilateral syndactyly of digits of her hands and feet, we took into consideration the possibility of oculodentodigital syndrome. Nonaka et al. [18] previously reported a case with similar findings to ours as oculodentodigital syndrome. However, our case was different from theirs because there was no finding of oculodentodigital syndrome except for the history of digital fusion.

In summary, we could not diagnose this case as first and second branchial arch syndrome. However, we could not rule out this syndrome, because of suspicious findings as well as the impairment of her left ear.

## Figures and Tables

**Table 1:** Cephalometric analysis.

	Patient	Mean ± SD
Facial angle	87.7	83.2 ± 2.9
Convexity	7.0	9.5 ± 4.4
A-B plane	−0.7*	−6.2 ± 2.7
Mandibular plane	25.6*	34.0 ± 3.8
*y*-axis	64.6	66.2 ± 3.0
Interincisal angle	141.7*	118.7 ± 7.5
L-1 to mandibular	89.3	95.4 ± 6.3
SNA	84.2	81.5 ± 4.2
SNB	82.6	77.1 ± 3.8
U-1 to SN	96.5	105.4 ± 5.2
Gonial angle	118.8*	131.0 ± 5.6

Asterisks indicate the out of range of 2SD limits.
